# The Causes of Dental Implant Migration into the Maxillary Sinus: A Case Series Study from 25 Years of Experience

**DOI:** 10.30476/dentjods.2023.95807.1898

**Published:** 2024-03-01

**Authors:** Hamid Mahmood Hashemi, Saba Mohammadi, Farnoosh Razmara

**Affiliations:** 1 Dept. of Oral and Maxillofacial Surgery, School of Dentistry, Tehran, University of Medical Sciences, Tehran, Iran; 2 Craniomaxillofcial Research Center, Tehran University of Medical Sciences, Tehran, Iran; 3 Dentist, School of Dentistry, Tehran University of Medical Sciences, Tehran, Iran

**Keywords:** Maxillary sinus, Sinus Floor Augmentation, Dental Implants, Complications

## Abstract

The use of dental implants to restore edentulous jaws has become commonplace. Usually, in the maxilla, following a tooth extraction, the height of alveolar bone decreases. This alteration in bone increases the risk of implants migrating into the sinus. In general, Caldwell-Luc and endoscopic surgery are performed to retrieve dental implants. In this case series, we collected data from 39 patients who had the complication of implant displacement within the maxillary sinus for 25 years. All the implants were removed using the Caldwell-Luc technique. Implant migration happened following functional loading, during the prosthetic procedure, due to lack of osseointegration in 3 patients, and during implant placement into the fresh socket in 3 patients. In the remaining cases, migration occurred preoperatively or postoperatively and prior to implant loading. Insufficient bone quantity is sometimes causing the implant to migrate to the maxillary sinus. In case of minimal bone height, a sinus lift before implant placement should be conducted. Retrieval of an implant pushed inside the maxillary antrum using the Caldwell Luc approach proved to be a reliable technique.

## Introduction

Tooth loss or extraction in the posterior part of the upper jaw due to trauma, malformation, periodontal disease, and dental caries leads to edentulism. Edentulate area in the molar and premolar segment of the upper jaw gives rise to a decrease in bone mass and causes bone resorption [ 1 ]. Dental implant placement in the edentulate areas is the treatment of choice to restore function and esthetics [ 2 ]. This procedure is safe and has an excellent prognosis [ 3 ]. Panoramic and cone-beam computed tomography (CBCT) scans before implant insertion are imperative to examine residual alveolar bone volume, features, and landmarks [ 4 ]. If the height of the alveolar ridge is inadequate, sinus elevation should be performed through a sinus lift to enhance the amount of bone in the upper jaw [ 3 ]. Failure to appropriately plan and assess the bone prior to implant surgery may result in complications such as dental implant dislocation within the sinus [ 5 ]. Migration of dental implants inside the maxillary sinus can induce severe effects, including sensory disorder, sinusitis, and oroantral fistula. The dislocated implant can unsettle the anatomy of the sinus and disturb the mucociliary escalator in the sinus membrane [ 6 ]. Implant migration within the maxillary sinus might occur during implant placement or postoperatively, during osteointegration, after implant load, or during prosthetic restoration. Maxillary sinus pneumatization, extremely resorbed bone, excessive bite force, bruxism, absence of primary stability, inexperienced surgeon, over-drilling, intense tapping, peri-implantitis bone loss, and considerable pressure upon nonintegrated implant removal may result in implant push inside the sinus [ 6 ]. An instant removal of dislocated implants from the maxillary sinus is generally suggested. If retrieval of the migrated implant from the maxillary sinus is postponed, it is necessary to control the sinusitis to avert further complications [ 6 ]. Surgical approaches for shifted dental implants within the maxillary sinus involve the Caldwell-Luc antrostomy and Endoscopic sinus surgery [ 6 ]. The Caldwell-Luc procedure uses an intraoral approach through a bony window in the maxillary sinus lateral wall. Endoscopic surgery utilizes transnasal access to enter the sinus throughout the maxillary ostium in the middle meatus [ 7 ]. The Caldwell Luc procedure has been recommended to retrieve dental implants from the sinus. The Caldwell Luc surgical technique offers an enhanced visualization of the operation area, facilitated approach to the site, and can be done under local anesthesia [ 8 ]. Despite that, endoscopy surgery is conducted under general anesthesia, requires more expensive equipment, and has a long operation time [ 9 ]. This paper aims to present the causes of displaced dental implants in the maxillary sinus. 

## Case Presentation

The study protocol was approved by the Ethics Committee of Tehran University of Medical Sciences (TUMS); IR.TUMS.DENTISTRY.REC.1401.036

### Clinical materials

**Figure 1:**
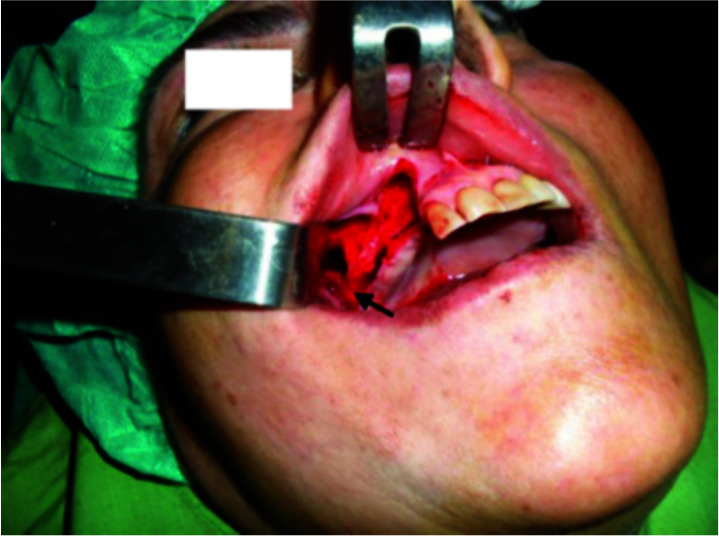
Caldwell-Luc incision

In 25 years between 1996 and 2021, 39 patients (21 females, and 18 males) were referred to a private dental clinic by general dentists, in result of dental implant displacement into the maxillary antrum. The age range was 28-73 years in females and 30-70 years in males. Caldwell Luc’s surgical technique (Figure 1), under local anesthesia, was chosen to extract dental implant from the maxillary sinus. All surgical procedures were conducted by a single proficient surgeon. 

### Preoperative evaluation, surgical procedure, and follow-up

On the day of procedure, a panoramic radiograph and CBCT were used to locate migrated dental implant. Preoperative medication regimen in 48 hours before included: tablet Co-amoxiclav 625mg 3 times a day [ 10 ] and mouth rinsing before surgery with 0.2% chlorhexidine gluconate (CHX). All patients underwent local anesthesia. After that, osteotomy with dental carbide bur #5 under constant irrigation with normal saline was performed to create a bony window in the lateral wall of the maxillary antrum. Following the bony window creation, an incision was made in the sinus membrane to approach the maxillary sinus and retrieve the dental implant. Through this surgical opening dental implant was retrieved by suction or rongeur. Then the flap was sutured by using 4-0 vicryl thread sutures. Post-surgery antibiotics (for one week), nasal spray decongestant, and analgesic were prescribed for patients to prevent sinus infection. Correspondingly, for post-surgical care, patients were advised in order to avoid nose-blowing for two weeks and to clean the area with 0.2% CHX solution, two times daily during seven days. 

## Results

**Figure 2:**
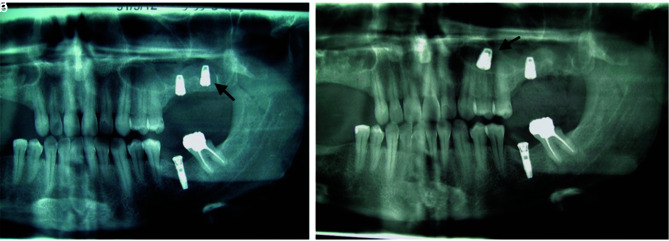
Poor bone quality and quantity

**Figure 3:**
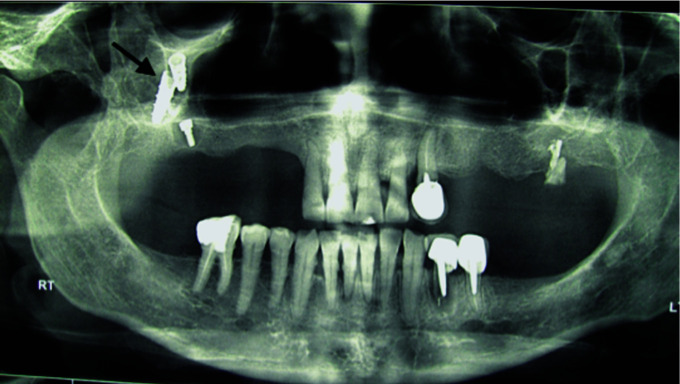
Implant displacement into the maxillary sinus during healing abutment placement

**Figure 4:**
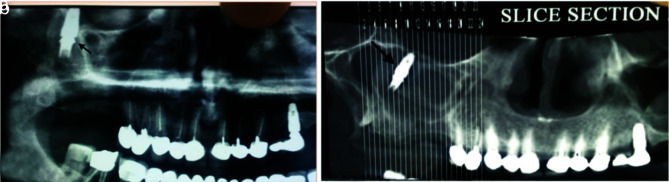
Implant displaced into the sinus during impression making

From 39 patients, the displacement has been occurred during implant placement in 22 patients. It was as a result of inadequate bone height (Figure 2) in 11 patients (6 male, and 5 female) and due to over drilling and wide socket in 11 patients (4 male, and). In six patients (2 male, and 4 female), implant migration has been occurred during closed sinus lift procedure by crestal approach. For five patients (3 male, and 2 female), displacement was happened during the placement of healing abutment due to lack of osseointegration (Figure 3). In three patients (1 male, and 2 female), dislocation occurred during prosthetic procedures caused by inadequate osseointegration (Figure 4). Migration occurred during the implant insertion into fresh extraction socket in three patients (2 male, and 1 female) (Table 1). Eighteen women and thirteen men were reluctant towards implant placement after displacement occurred however, the remaining patients accepted resumption of reconstruction and implant placement. Up to now, no complications occurred during the 2-10-year post-surgery follow-up duration.

**Table 1:** Reasons for implant migration into the sinus

Stage/ reason of displacement		Gender	
		Male	Female
Intraoperation	Immediately implantation	2	1
	Inadequate bone height	6	5
	Closed sinus lift procedure	2	4
	Over drilling	4	7
Post operation	Placement of healing abutment	3	2
	Prosthetic procedures	1	2

## Discussion

To prevent displacement of the implant into the maxillary sinus, we should abide by some principles. Dentists must be aware of this operation and capable of managing these complications. Before retrieval of the dislocated dental implant out of the maxillary sinus, accurate radiographic assessment is required to determine the exact location of the implant [ 9 ]. Migration of the implant into the maxillary sinus can happen intraoperatively or postoperatively whether before implant loading or after functional loading [ 11 ]. Intraoperative displacement of dental implants into the sinus may be due to implant placement in regions with low quality and quantity of alveolar bone, inexpert surgeon, over-preparation of the implant site, applying heavy pressure within implantation, and sinus perforation while drilling [ 4 , 11 - 12 ]. All these conditions lead to poor primary stability of the implant, notably the major reason of implant displacement during this point [ 11 ]. Inadequate initial stability leads to micromotion of dental implants, which prevents blood clotting process and revascularization and therefore obstructs ossification [ 11 - 12 ]. Displacement of dental implant postoperatively before implant loading can be due to loss of osseointegration because of existing bone infection, which leads to bone destruction, and osteoporosis or osteopenia resulting in bone deterioration [ 11 ]. Implant migration after functional loading includes erratic masticatory force, exertion of destructive forces on the surrounding bone, and implant loading not more than 3 weeks after placement [ 11 ]. Two principal management methods have been conducted to extract shifted implant from the sinus. One of them is the Caldwell-Luc procedure with intraoral access, which is conducted through the formation of a window in the anterolateral wall of the sinus. The other one is endoscopic sinus surgery, which is operated through a transnasal approach [ 3 ]. Caldwell-Luc operation is suggested for foreign body retrieval, repairing the oro-antral fistula, failing in endoscopic sinus surgery, and mucous cell metaplasia [ 9 ]. In our case series, we used Caldwell-Luc operation to remove dislocated maxillary implants. Juceléia Maciel *et al*. [ 8 ] described a case with shifted implant inside the sinus due to insufficient bone height between the crest of the alveolar ridge and the floor of the maxillary antrum during implantation. The migrated implant was removed by using the Caldwell-Luc surgical approach. Mumtaz *et al*. [ 3 ] presented a case of a man with the chief complaint of headache and nasal blockage for one year due to displacement of implants within the maxillary sinus. The patient underwent extraction of the implant through Caldwell-Luc technique. Yifat Manor *et al*. [ 7 ] reported 55 cases of implant dislocation within the sinus. Prior to implant placement, sinus augmentation was performed in 46 patients. In 52 cases, migrated implant was extracted using the Caldwell-Luc technique. It has been concluded that sinusitis and oroantral communication were the most complications among cases following implant displacement. Zaid Hamdoon *et al*. [ 13 ] reported 11 patients with implant migration into the sinus. Different surgical approaches were conducted to remove dental implants from the sinus. The surgical approach in three patients was the anterior-lateral window technique or Caldwell-Luc approach. According to this study, the least challenging approach was the Caldwell-Luc technique. Nicola Sgaramella *et al*. [ 4 ] reported 21 cases of implant displacement (24 implants). Implant displacement occurred after operation preceding implant load for ten implants. Dislocation of Implant in one case occurred after functional loading. Caldwell-Luc surgery was performed for eight patients. This procedure is the gold standard to enter the maxillary sinus regarding mucosal regeneration, better healing, less inflammation, performance under local anesthesia, and less complication in comparison with endoscopic surgery [ 13 ]. Insufficient bone height as a result of sinus pneumatization and alveolar bone loss at the site of implant placement is the leading reason for displacement of dental implant. When bone height is less than 5mm, sinus lift surgery should be conducted to prevent implant failure and migration [ 14 ]. Nicola Sgaramella *et al*. [ 4 ] reported 21 cases in which bone height was <5 mm at 19 implant sites of out 24 sites. Based on existing radiographic images, most of our patients demonstrated inadequate bone height, which required a sinus lift procedure before implantation. Despite that, no sinus augmentation was performed for them. Seemingly, Nicola Sgaramella *et al*. [ 4 ] reported 21 cases of implant dislocation, which sinus lift surgery was not conducted in 15 patients. Most of the cases with implant displacement were that of male participants. This finding is in accordance with Mumtaz *et al*. [ 3 ], Yifat Manor *et al*. [ 7 ], and Zaid Hamdoon *et al*. [ 13 ] and in contrary with Nicola Sgaramella *et al*. [ 4 ]. The movement of dental implant inside the maxillary sinus leads to reluctance for further implant placement in the patients who experienced the failure in their first implant surgical attempt. Therefore, it is advisable to have an appropriate pre-operation evaluation of bone condition to prevent implant displacement and motivate patients to complete the treatment. Among our cases, more specifically, 13 men and 18 women out of 39 patients were reluctant towards implant placement after displacement occurred. This complication led to a reduction in tendency for implant placement in patients and their families. So far, a study like the present study has not been done in different stages of implantation in this field. Additionally, the extended post-surgery follow-up period is notable feature of this study. Informed consent was obtained from all the reported patients.

## Conclusion

A very good pre-operative assessment of the bone should be conducted to prevent such complications. In case of minimal bone height, a sinus lift procedure before dental implant placement should be conducted to increase bone quantity and quality. A clinician should be certain about its primary stability when inserting the implant, also during impression the implant must have enough osteointegration. Retrieval of a displaced dental implant within the maxillary sinus through Caldwell-Luc surgical approach proved to be a reliable technique with fewer complications. 
